# Water Breakup at Fe_2_O_3_–Hematite/Water Interfaces: Influence of External Electric Fields from Nonequilibrium *Ab Initio* Molecular Dynamics

**DOI:** 10.1021/acs.jpclett.1c01479

**Published:** 2021-07-16

**Authors:** Zdenek Futera, Niall J. English

**Affiliations:** † Faculty of Science, University of South Bohemia, Branisovska 1760, 370 05 Ceske Budejovice, Czech Republic; ‡ School of Chemical and Bioprocess Engineering, University College Dublin, Belfield, Dublin 4, Ireland

## Abstract

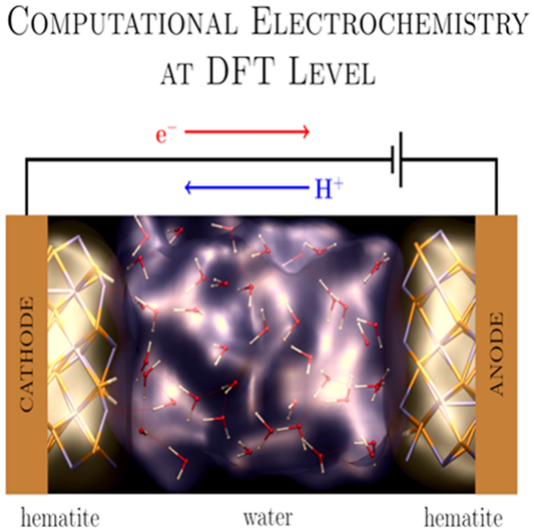

The dynamical properties of physically and chemically adsorbed water molecules at pristine hematite-(001) surfaces have been studied by means of nonequilibrium *ab initio* molecular dynamics (NE-AIMD) in the *NVT* ensemble at room temperature, in the presence of externally applied, uniform static electric fields of increasing intensity. The dissociation of water molecules to form chemically adsorbed species was scrutinized, in addition to charge redistribution and Grotthus proton hopping between water molecules. Dynamical properties of the adsorbed water molecules and OH^–^ and H_3_O^+^ ions were gauged, such as the hydrogen bonds between protons in water molecules and the bridging oxygen atoms at the hematite surface, as well as the interactions between oxygen atoms in adsorbed water molecules and iron atoms at the hematite surface. The development of Helmholtz charge layers via water breakup at Fe_2_O_3_–hematite/water interfaces is also an interesting feature, with the development of protonic conduction on the surface and more bulk-like water.

The study of the properties of aqueous solutions in contact with metal–oxide interfaces has undergone a substantial increase in scrutiny in recent years, often to exploit support-metal–support-interaction (SMSI) modification of catalytic properties.^1^ Although TiO_2_ is one of the most studied metal oxides in the literature, α-Fe_2_O_3_ has come to be of equal, if not more, interest in recent years, due to its relative ubiquity and low cost.^2−10^ However, despite recent progress in our understanding of titania–water interfaces,^11,12^ including via theoretical and molecular simulation methods,^13^ the successful modeling of hematite–water interfaces remains rather challenging and elusive. Such metal–oxide/water interfaces provide a rich environment for the study of the dynamical properties of confined water molecules; this is particularly so where hydrogen-bonded molecules play an important role in stabilizing solutes via solvent interactions and in forming “cages”. Molecular dynamics (MD) has been useful to some extent in characterizing the dynamical and vibrational behavior of adsorbed water molecules on metal oxide surfaces, e.g., titania–water,^14−23^ given titania’s status as the putative, or ideal, aspirant “prototype” material for solar water-splitting. In the case of titania–water, these studies have included orientations of surface water dipoles,^15^ their vibrational spectra,^16^ the kinetics of their hydrogen bonding arrangements,^17^ ion adsorption,^18^ electric double layer structure,^19−21^ surface-protonation effects,^22^ and spatial distribution functions.^23^ In terms of density-functional theory (DFT) level calculations for titania–water interfaces, particularly from *ab initio* MD (AIMD), a range of surfaces have been simulated from partial to full coverage, and multiple water layers.^13,24^ The influence of defects on anatase-101 surfaces on water adsorption energies has been studied at defective sites.^25^ Hydroxide ions have also been studied at these surfaces.^26^ Car–Parrinello MD (CPMD) simulations of pristine and defective anatase-101 surfaces have been performed with one, two, and three layers of water adsorbed thereon,^27^ with other CPMD work leading to a different surface reconstruction of rutile-011 than under vacuum or low-coverage conditions.^28^ AIMD has also offered key insights into the librational motion of higher-frequency modes of water adsorbed to titania in recent studies.^29,30^

The application of MD, whether using classical pairwise potentials or DFT, to interfaces between iron oxide and water has been less studied, despite the potentially promising prospect of iron oxide for catalytic reactions and water-splitting. More specifically, 001-surface hematite has been found experimentally to be stable and usually exposed in natural crystals.^31,32^ Rohrbach et al.^31^ and Bergermayer et al.^32^ have used DFT to characterize the structure and composition of these (dry) surfaces. Yin et al.^33^ and Trainor et al.^34^ have applied DFT to study the adsorption of water on hematite surfaces, and these are in agreement with the experiment which shows that hydroxylated terminations are more stable. Kubicki et al.^35^ have examined (010) surfaces of goethite (α-FeOOH) using the PBE functional, paying particular attention to surface complexation models as a function of solvation. Nguyen et al.^36^ have improved the DFT treatment of hematite–water interfaces by use of PBE+U. In many cases, very low energy barriers were found for water dissociation on hematite (001) surfaces, confirming experimental reports of widespread room-temperature dissociation of water on hematite (001);^36^ this is in some contrast to the debate as to the dominance of physical or chemical water adsorption on rutile (110) at room temperature.^37−39^ AIMD studies of the water–hematite interface tend to be reported more rarely than those for water/titania.^2^ Arguably, despite encouraging recent progress in reactive force-field simulations,^40,41^ AIMD is needed to capture the rich physical complexity of room-temperature chemical adsorption and the dynamical properties of the surface hematite layers, along with those of the water. Certainly, the relatively low energy barriers for water dissociation indicated by Nguyen et al.^36^ suggest that water dissociation may be observable over picosecond time scales in room-temperature AIMD; this was indeed the case in ref (2).

Bearing this need for AIMD in mind to capture the complexity of water/hematite interfaces, the present study wishes to establish the effect of externally applied electric fields utilizing nonequilibrium AIMD (NE-AIMD) simulations of α-Fe_2_O_3_ (001) surfaces in contact with (initially) physically adsorbed, full bulk layers of water (as opposed to a somewhat artificial simple water monolayer or bilayer). This is of essential relevance in electrochemistry and electro-catalysis, in enhancing our underlying molecular-level knowledge of electrical biases applied to water/metal–oxide interfaces.^42^ Indeed, this has been made possible recently by the advent of long-time NE-AIMD in external electric fields by a combination of the Berry-phase approach^43^ with second-generation Langevin dynamics,^44^ applied with success to bulk water for both approaches in tandem.^45^ Indeed, the Berry-phase approach has been applied previously, with success, toward water molecular breakup with standard AIMD also in different environments.^46−50^ The essential goal of the present work is to assess, *inter alia*, the nature of water (electro-) dissociation at water/hematite interfaces. The evolution of chemical and physical adsorption behavior, protonic conduction in surface-bound and more bulk-like water layers, together with the development of a surface electric potential and Helmholtz layer, are important observables. It must be noted that the present NE-AIMD simulations describe *electronically ground-state* (electro-) dissociation.

(NE-)AIMD simulations under three-dimensional periodic boundary conditions (PBC) were performed for a condensed state of liquid water over a mobile slab of pristine, initially anhydrous hematite-(001) Fe_2_O_3_ surface. This surface was cleaved as a 2 × 1 × 1 orthorhombic simulation cell (*x* = 10.076 Å, *y* = 8.726 Å, *z* = 13.772 Å) from bulk α-hematite (space group 167, *R*3*c*) to yield a nonpolar and dipole-free surface, and the direction of heterogeneity was the *z*-axis (cf. Figure 1). The hematite contained 120 atoms. 42 liquid-state water molecules were placed at a density of approximately 1 g/cm^3^ in contact with the hematite, with the same *x*–*y* simulation cell cross-section and a water-layer thickness of ca. 14 Å. The layer in contact with the hematite was adsorbed physically, using a motif from molecular mechanics energy-optimization at 0 K with the SPC/E water model^51,52^ and a rigid hematite slab with charges of +1.5 e on the iron ions and −1 e on each oxygen ion.

**Figure 1:**
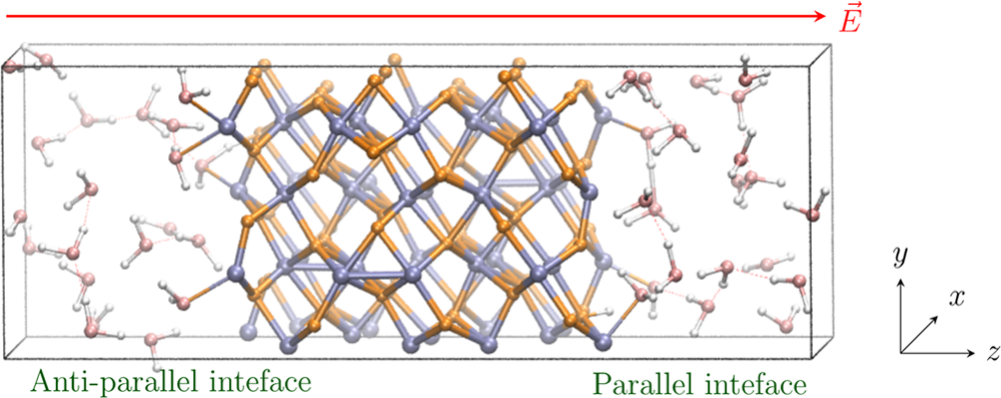
Model of the aqueous hematite interface used for the calculations. The hematite slab is located in the central part of the supercell (Fe shown in light blue and O in orange). Water molecules (pink O and white H) fill the rest of the simulation box on which the periodic boundary conditions are imposed. The direction of the applied static electric field *E* is indicated by the red arrow. The left-hand side (LHS) is the “lower” surface, whereat the external field vector is in antiparallel alignment vis-à-vis its local surface normal, while the opposite is the case on the “upper” surface on the RHS, with parallel field-vector alignment.

The DFT itself used the vdW-DF functional with the explicit nonlocal Dion–Rydberg–Schroder–Langreth–Lundqvist (DRSLL) correlation correction describing the van der Waals interaction.^53,54^ For the exchange part, the optimized Becke 88 (optB88) generalized gradient approximation (GGA) type functional was used.^55^ The DRSLL approach has been chosen due to its good description of water and its dynamical properties^56−61^ even in nonequilibrium simulations affected by electric fields.^45^ Γ-point sampling was used, given the reasonably large size of the periodic system, and water layer in the *z*-direction. The plane wave cutoff energy was set to 400 Ry, following convergence checks. Prior to AIMD and after molecular-mechanics optimization, both the atomic positions and lattice parameters were partially optimized using the vdW-DF method until the atomic forces were smaller than 0.02 eV/Å.

Now, as mentioned previously, Nguyen et al. have improved quantitative DFT simulation of hematite–water interfaces via GGA+U, finding, in particular, lower energy barriers for water dissociation.^36^ However, in the present work, we seek to gain qualitative insights into the basic mechanisms of (field-mediated) water dissociation; should the underlying (zero-field) dissociation-energy barrier be lower (as with PBE+U^36^), it would be all the more difficult to distinguish field-mediated intramolecular dissociation from that intrinsically present within the GGA+U framework’s lower energy barriers. Therefore, not without some reluctance, we opted for vdW-DF without the Hubbard U term in the present work, to allow for a crisp mechanistic distinction between field-induced breakage and underlying dissociation.

For NE-AIMD-propagation in external electric fields, in order to isolate athermal effects as much as possible from thermal effects, nonequilibrium (N)NVT^62,63^ was carried out. Long 50 ps NE-AIMD trajectories were performed in the presence of external fields, via second-generation Car–Parrinello MD;^64^ ref (64) indicates good structural and dynamical characteristics of water, e.g., radial distribution functions, self-diffusion coefficients, and vibrational spectra. A time step of 1 fs was used in conjunction with a Langevin stochastic thermostat for NE-AIMD, as well as for zero-field, equilibrium AIMD.^44,64^ NE-AIMD was performed using in-house-modified CP2K software.^45,65^ The wave function was described by DZVP basis set with core–electron GTH pseudopotentials from the CP2K database.

Uniform, static electric fields *E* were applied along the laboratory + *z*-direction via the Berry-phase approach (cf. Figure 1).^43^ The field strengths ranged up to 0.1 V/Å. Generally, in the condensed phase, water has intrinsic electric-field intensities in the range ∼2–3 V/Å,^42,62,63^ so the external-field torques on each molecule are of the order of 2–15% of those inherently present due to interaction with neighboring molecules, affording a reasonable “signal-to-noise” ratio and allowing for transition into the nonlinear response régime at ∼0.05–0.1 V/Å.^63^

As noted above, we use the supercell approach with periodic boundary conditions imposed in all three directions to simulate the aqueous hematite interface in the framework of DFT. The supercell, containing an equilibrated representative structure from the 100 ps equilibrium AIMD where no external electric field was applied (further referred to as “zero-field” simulations), is shown in Figure 1. The supercell was 9.743 × 8.438 × 27.023 Å^3^ in size, accommodating 24 Fe_2_O_3_ units in the hematite-(001) slab and 42 water molecules. In the beginning, the system was carefully equilibrated from its initial structure, which was optimized first and then slowly heated to a target of 330 K, which is typically used to simulate the room-temperature properties of liquid water within DFT.^45,66^ Then, the parameters of the Langevin thermostat, which was used together with the ASPC corrector^67^ in the second-generation CPMD procedure,^44,64^ were optimized. The Langevin parameters γ_*L*_ = 10^–2^ fs^–1^ and γ_*D*_ = 10^–4^ fs^–1^ were applied to provide numerically stable simulations with a long 1 fs time step. This setup was then used to study the water dynamics and electric-field effects thereon, with AIMD simulations under both equilibrium (zero-field) for 100 ps and in-field (NE-AIMD) conditions for 50 ps.

Although the initial structure was prepared in a way that the first solvation layers of the hematite surfaces were only weakly interacting with the oxide, i.e., physi-sorbed, the water molecules from these layers quickly adsorb chemically to the nonsaturated surface iron atoms, as was observed also in zero-field simulations in ref (2) for a very similar system. On average, four water molecules chemi-sorbed to each of the two hematite surfaces during zero-field equilibration, and even the spontaneous dissociation of one of them to H^+^ and OH^–^ species was observed. While the hydrogen proton moves to the nearest surface oxygen which is highly nucleophilic, the negatively charged hydroxyl group stays attached to the surface iron (cf. Figure 1). On the one hand, the protons are known to form rather complicated binary patterns on the hematite surface, and due to their small size, they can flip between outward- and inward-pointing directions, which affects the surface acidity.^68,69^ On the other hand, hydroxyl groups are less mobile, stabilized not only by interactions with the surface, but also by relatively strong hydrogen bonding with the water molecules from the first two solvation layers. This behavior reflects the strong water-splitting activity of the (001) hematite surface, and it is in agreement with previous experimental and computational observations.^2^

When the static electric fields of 0.05, 0.075, 0.0857, and 0.1 V/Å magnitudes were applied to the equilibrated system in the direction perpendicular to the hematite surfaces (cf. Figure 1, along the laboratory + *z*-axis), and the systems propagated further under NE-AIMD, expected^2,45,62,63^ partial dipole-alignment response of the water molecules occurred. This amplitude-dependent dipole alignment took place alongside concomitant reorientations, and this is consistent with our previous observations of this in NE-AIMD simulation of bulk water in external electric fields.^45^ Here, at hematite interfaces, the applied fields perturb the force balance between the surface and its first solvation layer. The interfacial water network, confined by relatively strong hydrogen bonding and electrostatic interactions, becomes disrupted—thereby accelerating the water-splitting reactions. These effects are strongest when the external field is *effectively* applied *toward* the hematite surface (i.e., on the left-hand side of the Fe_2_O_3_ slab in Figure 1, with the direction of the field-vector arrow pointing toward the oxide—an “antiparallel” field alignment). This is as opposed to the applied field acting in a direction parallel to the outward surface normal (shown on the right-hand side of the oxide slab in Figure 1, dubbed a “parallel” field alignment). Noting this *de facto* “field-direction dichotomy” existentially present in all NE-AIMD simulation for both surfaces on each side, we may observe rich and important differences in local behavior for each interface. Indeed, in the latter (“parallel”) surface case (RHS), the water molecules are “dragged” further from the oxide, while in the former (i.e., antiparallel) case (LHS), the water molecules tend to rotate and point by their hydrogen atoms to the surface, as one can see in Figure 2: snapshots of both cases from NE-AIMD simulations affected by different external fields are shown there, when comparing Figure 2, parts a, c, e, and g, on the left (RHS, Figure 1, “parallel”) versus Figure 2, parts b, d, f, and h, on the right (LHS of Figure 1, “antiparallel”). Further details of snapshots are depicted in Figure S1 of the Supporting Information, over a whole suite of simulations. Naturally, the electric fields help to overcome (already-low)^2^ energy barriers for water splitting, as the water molecules’ protons are “pushed” toward an even more (field-mediated) highly reactive surface oxygen atoms.

**Figure 2:**
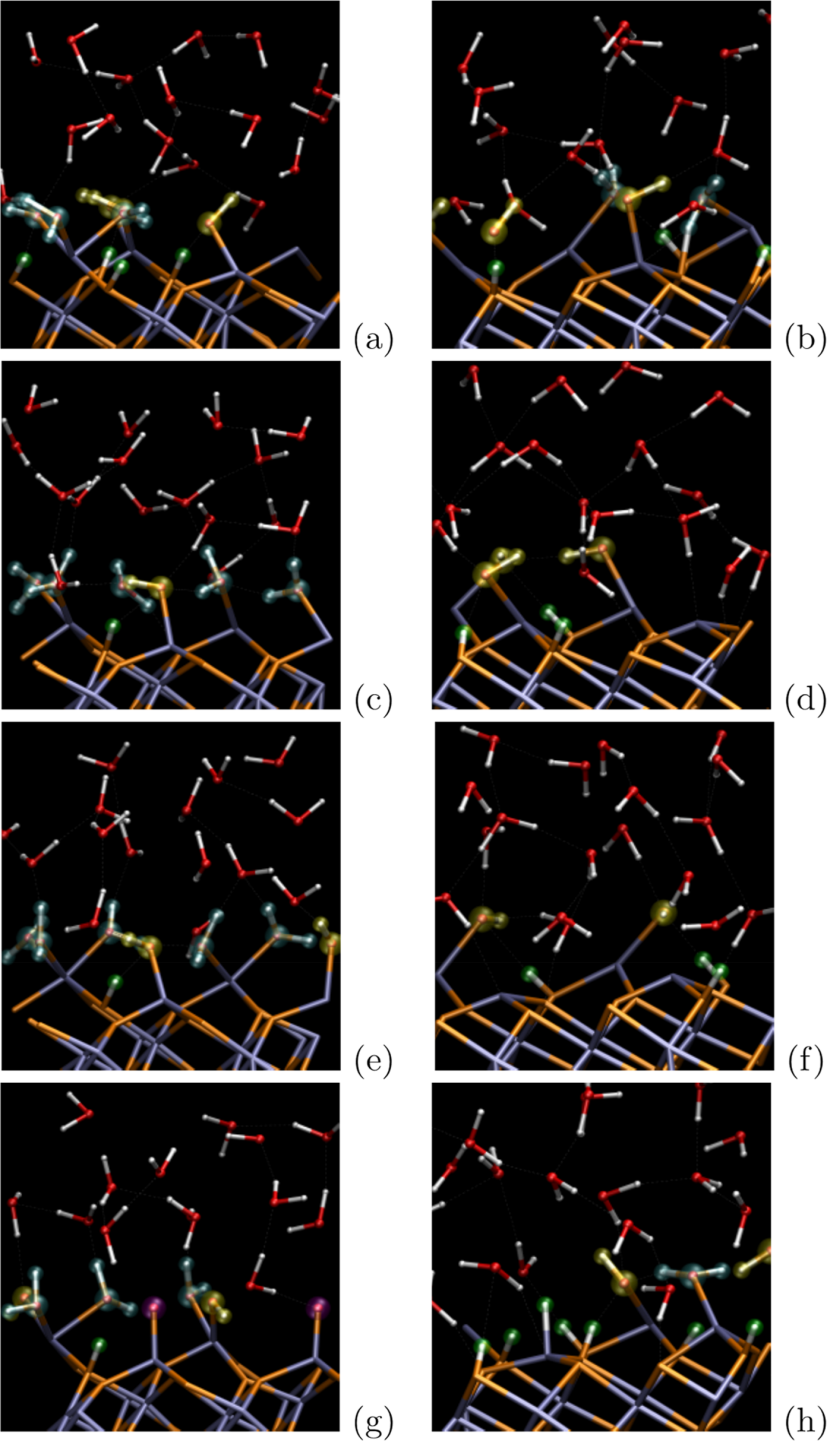
*Ab initio* molecular-dynamics snapshots of aqueous hematite interfaces under (a) zero-field conditions after 50 ps of production equilibrium AIMD, (c) static electric field of 0.05 V/A magnitude at 43.7 ps, (e) field of 0.075 V/A at 49.6 ps, and (g) field of 0.1 V/A at 25.7 ps where the applied field is parallel with hematite-surface normal (i.e., RHS of Figure 1). The complementary interfaces for local antiparallel external-field alignment are shown in panels b, d, f, and h, respectively (cf. LHS of Figure 1). The hematite slab’s Fe atoms are shown in light blue and oxygen atoms in orange. Adsorbed nondissociated water molecules are highlighted by cyan color, while dissociated hydrogen protons, hydroxyl groups, and oxygens adsorbed to the surface are depicted in green, yellow, and magenta, respectively.

Although the protons (electro-) released from water are stabilized on the negatively charged surface of hematite (especially for antiparallel external-field application), the hydroxyl group interaction with the iron is weakened as the protons (as opposed to entire “parent” water molecules of which they were formerly part) are “dragged” toward the bulk-water region by the applied fields. This proton-drift effect can be seen dramatically in changes of the water–oxygen density near the solid surfaces shown in Figure 3 and also in pair radial distribution functions in Figure 4—where individual interactions between surface atoms and water molecules are detected clearly. As a rather stark result, the first density peak located at ∼1.2 Å above the average position of the surface iron atoms is shifted to 2 Å, or even further, as the water molecules reorient markedly in the antiparallel field-application case (for example, see the LHS of Figure 1). However, resultant hydroxyl chemi-sorption to the nonsaturated surface iron atoms is strong, and tends to inhibit their desorption.

**Figure 3:**
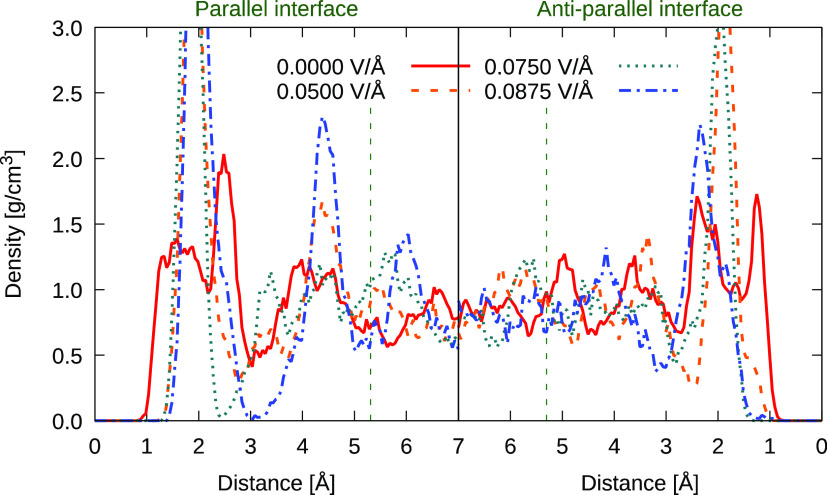
Density profile of water oxygen O_w_ atoms in the interfacial region (marked by vertical dashed lines) of hematite affected by the external static electric fields of magnitudes 0.05, 0.075 and 0.0875 V/Å applied in the direction perpendicular to the hematite surface. Densities at the interfaces where the surface normal is parallel (cf. RHS of Figure 1) are shown in the left panels here, while for antiparallel alignment in the right panel here, the reader is referred to Figure 1 (LHS). The zero-field density distribution (solid red line) is shown as a reference.

**Figure 4:**
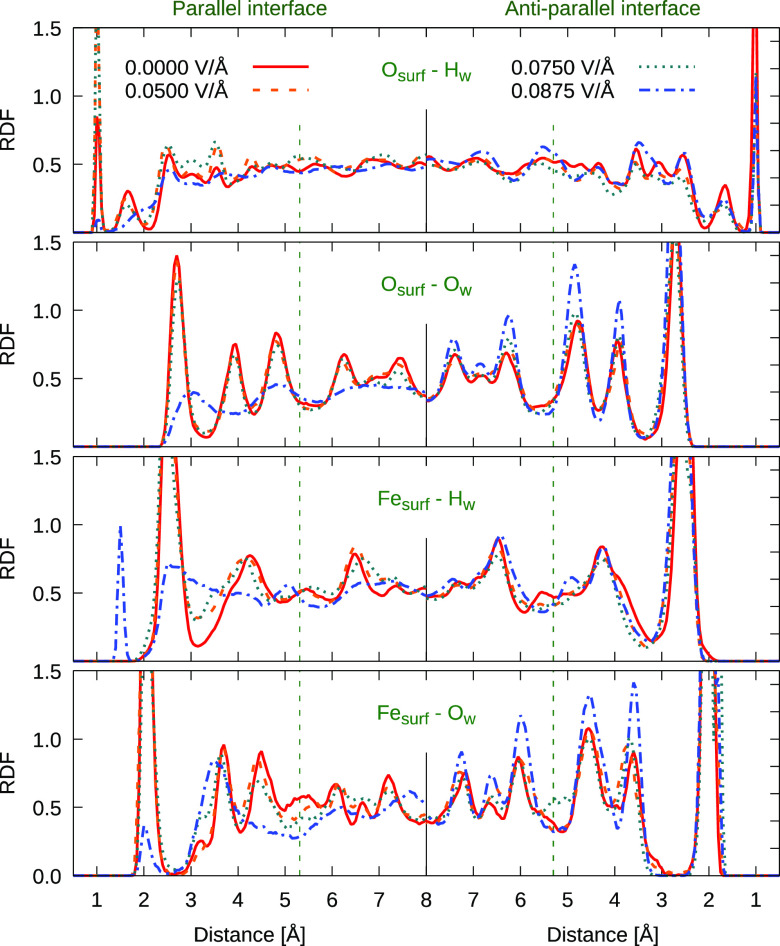
Radial distribution functions (RDF) detecting interactions of the hematite surface iron (Fe_surf_) and oxygen (O_surf_) atoms with the water oxygen (O_w_) and hydrogen (H_w_) as the external static electric fields of magnitudes 0.05, 0.075, and 0.0875 V/Å are applied in the direction perpendicular to the hematite surface. As in Figure 3, RDFs at the interfaces where the surface normal is parallel (cf. RHS of Figure 1) are shown in the left panels, while the case for antiparallel alignment is shown on the right panels (cf. LHS of Figure 1). The zero-field density distribution in red is depicted as a reference.

Probing further, striking differences emerge in the electric-field response of surface-adsorbed water molecules for the parallel/antiparallel field-application cases in the context of local-surface distributions of dipole-moment magnitudes of these molecules, and this is readily apparent in Figure 5. Under zero-field conditions, there are clear two-peak dipole distributions, distinguishing water molecules from the second and further solvation layers, with a molecular dipole moment of ∼1.5 D in the bulk-like region in the middle of the supercell, and water molecules from the first solvation layer which are strongly interacting with the surfaces, and exhibiting a substantially smaller dipole moment ∼1.1 D due to the relative depolarizing effect of the surface. The applied field then stabilizes the first hydration layer in the case of (local-surface) parallel-field application (cf. Figure 1, RHS interface), representing a *de facto* positively charged anode. In distinct contrast, the applied-field torque reorientating the water molecules at the interface in the case of antiparallel field application (Figure 1, LHS) serves to weaken substantially their interaction with the surface—resulting in (partial) desorption, somewhat less surface depolarization, and a consequential increase of the molecular dipole moment. The desorption is also detectable in changes in the lengths and angles of hydrogen bonds between the surface oxygen atoms and water (cf. Figures S3 and S4, Supporting Information). Similar external-field effects, in terms of applied-field orientation and adsorbed-water dipolar behavior, were observed in empirical and ReaxFF simulations for aqueous interfaces of titania.^40,42^

**Figure 5:**
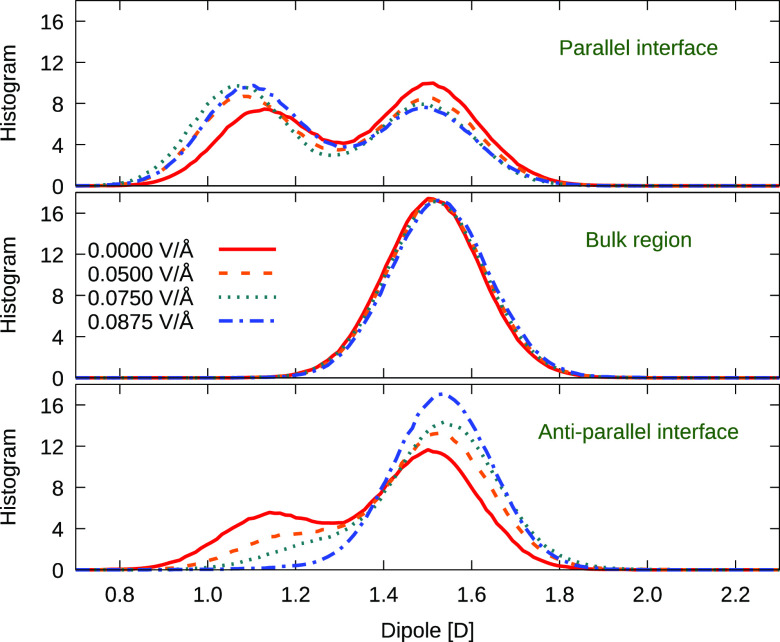
Changes in the nondissociated water-molecule dipole distributions induced by the external static electric fields of magnitudes 0.05, 0.075, and 0.0875 V/Å applied in the direction perpendicular to the hematite surface. Distributions at the interfaces with the surface normal (anti)parallel to the applied field direction (cf. Figure 1) are compared with the distributions collected in the bulk-water region where the water is not materially affected by interactions with the hematite. The zero-field distributions (solid red line) are shown as a reference.

When the external fields were relatively weak in our NE-AIMD simulations, i.e., up to 0.05 V/Å, the corresponding electric-field force is not strong enough to desorb the dissociated charged species and “drag” them toward the other (periodically imaged) surface, which represents the counter-electrode what amounts to our periodic-model “thought experiment” in electrochemistry. The interfaces, under these conditions, remain effectively charge-neutral, with the same number of adsorbed H^+^ and OH^–^ species—as we show in Table 1. However, as the field magnitude increases to 0.075 V/Å, and higher, the protons start to be (electro-) released from the chemi-sorbed water molecules atop the positively charged, field-parallel surface (RHS, Figure 1) and they are transported relatively quickly to the other surface (LHS) by the well-known Grotthus hopping mechanism, through the network of hydrogen-bonded water molecules. This leads to charge separation, and the formation of Helmholtz layers at both interfaces. The protons so transported to the field-antiparallel surface (LHS, Figure 1) recombine with the OH^–^ groups, and they were reduced, poised to form molecular hydrogen at higher concentrations. At extreme conditions, when we applied the 0.1 V/Å field on the system (well into the nonlinear response régime),^63^ we observed the formation of an oxygen anion on the field-parallel (RHS) surface and both of the respective interfaces charged to ±5 *e* in our model (cf. Table 1). However, this extreme régime is already beyond the numerical-fidelity limits of the employed second-generation CPMD approach, in that the simulations become manifestly unstable after 10 ps (cf. Figure S2 in the Supporting Information) and we were not able to propagate the system further to observe tangible H_2_ formation. Therefore, at best, we must label these “H_2_-formation” hints as being very tentative, and possibly prone to artifact in an NE-AIMD sense, although indeed eminently plausible.

**Table 1:** Number of Protons (H^+^), Hydroxyl Groups (OH^–^), Oxygens (O^2–^) and Non-dissociated Water Molecules (H_2_O) Directly Adsorbed to the Aqueous Hematite Surface Found in MD Simulations Affected by the Static Electric Field of Specified Magnitude [V/Å] at a Given Time [ps] after equilibration^a^

		parallel interface					antiparallel interface				
field	time	H^+^	OH^–^	O^2–^	H_2_O	charge	H^+^	OH^–^	O^2–^	H_2_O	charge
0.0000	0.0	1	1	0	2	0	0	0	0	4	0
0.0000	50.0	4	3	0	3	1	3	3	0	2	0
0.0500	43.7	1	1	0	5	0	3	3	0	0	0
0.0750	49.6	1	2	0	5	–1	3	2	0	0	1
0.0875	38.3	1	2	0	3	–1	2	1	0	0	1
0.1000	25.7	1	2	2	3	–5	7	2	0	1	5

a Formal charge [e] of the solvent part of the interfaces is given by the number adsorbed species. Interfaces where the applied fields are parallel/anti-parallel to the surface normal are compared (cf. Figure 1).

Finally, we calculated a mean electronic-density profile *n*(*z*) along the supercell, as well as the total charge density ρ(*z*) involving also the nuclear charges, in addition to the corresponding Hartree potential *V*_*H*_(*z*)—which is the *de facto* electrostatic potential that the electrons experience (or, in other words, “feel”). These quantities were obtained as averages over 100 samples per each trajectory under the influence of different external fields, and their profiles in the interfacial region between the hematite surface and bulk water are shown in Figure 6. The profile of the Hartree potential across the whole supercell can be seen in Figure S5 of the Supporting Information, where the large interfacial potential drop is noticeable. The potential difference between the bulk hematite and bulk water was found to be 10.5 V, which is consistent with mean-inner-potential (MIP) values previously published in the literature: the experimental MIP of liquid water is 4.5 V,^70^ while the values for hematite are estimated to be in a range between 16.2 and 19.5 V.^71^ Althought the potential values are very large, the external fields induce dramatic changes, in particular at the antiparallel interface, representing the positively charged anode. This is connected with proton adsorption and corresponding charge redistribution on these interfaces, in accordance with the previous discussion.

**Figure 6:**
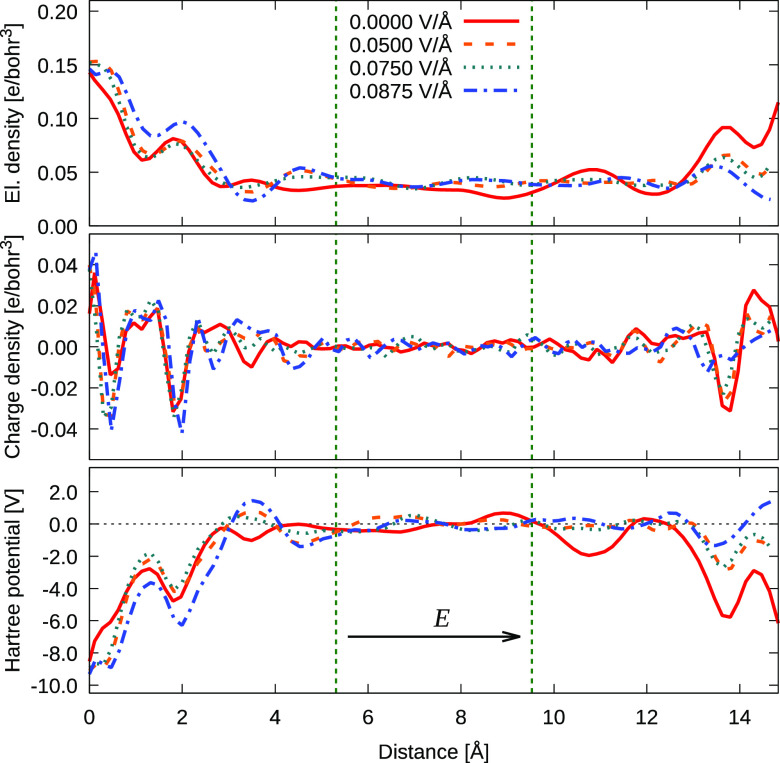
Electron density *n*(*z*), total charge density ρ(*z*), and Hartree potential *V*_*H*_(*z*) in the water region of the supercell averaged over (NE-)AIMD samples. The distance is measured from the average position of the outermost Fe-atom layer at the hematite surface. The vertical dashed green lines mark the *de facto* boundary of the interfacial region. Direction of the applied electric field is indicated by the black arrow. The Hartree potential is aligned to zero in the bulk-water region.

However, the full charge density is often represented by the atomic point charges *Q*_*i*_ to reduce size of the stored data and increase efficiency of already demanding first-principles calculations. The electrostatic-potential profile φ(*z*) can be then obtained by solving the one-dimensional Poisson equation
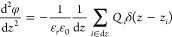
where the charge density ρ(*z*) is now approximated by a point-charge distribution, and ε_*r*_ represents a relative permittivity of the environment. If we, for simplicity, neglect variations of the permittivity in the interfacial region and used Hirshfeld atomic charges^72^ tracked along NE-AIMD trajectories, one obtains the charge density and potential profile as shown in Figure S6 of Supporting Information. The charge density is qualitatively similar to the mean total charge density obtained from DFT (cf. middle panel in Figure 6), where the considerable variations near the hematite surface reflect the water layering typical for aqueous/metal-oxide interfaces. The corresponding changes in intrinsic electric fields, varying between +2 and −3 V/Å, are comparable with results of empirical and ReaxFF force-field studies in the vicinity of hydrated titania surfaces.^40^ However, the resulting electrostatic-potential drop at the interface is approximately half of the Hartree-potential drop shown in Figure 6. The difference is a result of not just the monopole approximation, where the full-space charge density is described by set of discrete charges located at atomic nuclei. Indeed, since the Hartree potential describes the mutual electron–electron interaction, the potential constructed from point charges reflects also the interaction with atomic nuclei, as the point-charge distribution describes the total charge density and not just the electron density *per se*. However, the point-charge results need to be interpreted cautiously because they might lead to artifacts and nonphysical results, as was previously discussed by Kathmann et al. with acuity in ref (73).

In closing, we have investigated the catalytic activity of hematite (001) surfaces to act as surface catalysts in external-field-enhanced splitting of water, and simulated the electrochemical cell with applied static electric fields using DFT. Spontaneous molecular adsorption of water on the nonsaturated surface iron atoms was observed, followed by water dissociation, resulting in separated H^+^ and OH^–^ species attached to surface O and Fe, respectively. The charge separation continued until there was ∼50% coverage of the hematite surface, which is similar to the behavior of wetted titania.^40^ However, the charged species cannot spontaneously leave the surface and diffuse out of the interface region because of strong binding to the hematite and electrostatic stabilization, which prevents their desorption by a high energy barrier. Under zero-field (or, equivalently, open-circuit) conditions, these species form, *de facto*, the termination layer of the hematite.

Applied external electric fields, connected with a bias potential typically controlled in the electrochemical applications, act as a driving force to trigger H^+^/proton transport across the solution to the other electrode by a rapid Grotthus-hopping mechanism. In this way, the charged species become separated, forming Helmholz layers screening the surface charges of the electrodes. Although the small size of the model system necessary for efficient DFT calculations allows us to observe neither the buildup of the full electric double layer, with its diffusion-layer part, nor indeed the space-charge layer on the hematite surface itself, the function and effects of the applied static electric fields are clearly visible—often in dramatic fashion with opposing local directions perpendicular to each surface (cf. Figure 1: LHS for “toward”, RHS for “away from”). On the other hand, the potential bias *per se* affects band bending at the hematite surface region, and governs hole transfer in the water-splitting reaction. Although this process, in and of itself, is not studied here, it is inevitably and intimately connected with the well-known photoelectrochemical (PEC) catalytic activity of hematite and the general renewable-energy, CO_2_-lowering agenda.^74^

## Supporting Information Available

Representative snapshots of the system (Figure S1), potential-energy evolution (Figure S2), hydrogen-bond geometry (Figures S3 and S4), spatial Hartree-potential distribution (Figure S5), Hirshfeld-charge charge, field, and potential distributions (Figure S6) (PDF)
