# Supplementary material for: Water Breakup at Fe2O3–Hematite/Water Interfaces: Influence of External Electric Fields from Nonequilibrium Ab Initio Molecular Dynamics
Source: J Phys Chem Lett. 2021 Jul 16;12(29):6818–26. doi: 10.1021/acs.jpclett.1c01479 (PMC8397349; doi:10.1021/acs.jpclett.1c01479)
Supplement: Supplementary file 1 — jz1c01479_si_001.pdf [file jz1c01479_si_001.pdf]

Supplementary Information
for
Water Break-up at Fe_2O_3-Hematite/Water Interfaces: Influence of External Electric Fields from Non-equilibrium *Ab-initio* Molecular Dynamics

Zdenek Futera* [a] and Niall J. English * [b]

a) Faculty of Science, University of South Bohemia, Branisovska 1760, 370 05 Ceske Budejovice, Czech Republic.
b) School of Chemical and Bioprocess Engineering, University College Dublin, Belfield, Dublin 4, Ireland.

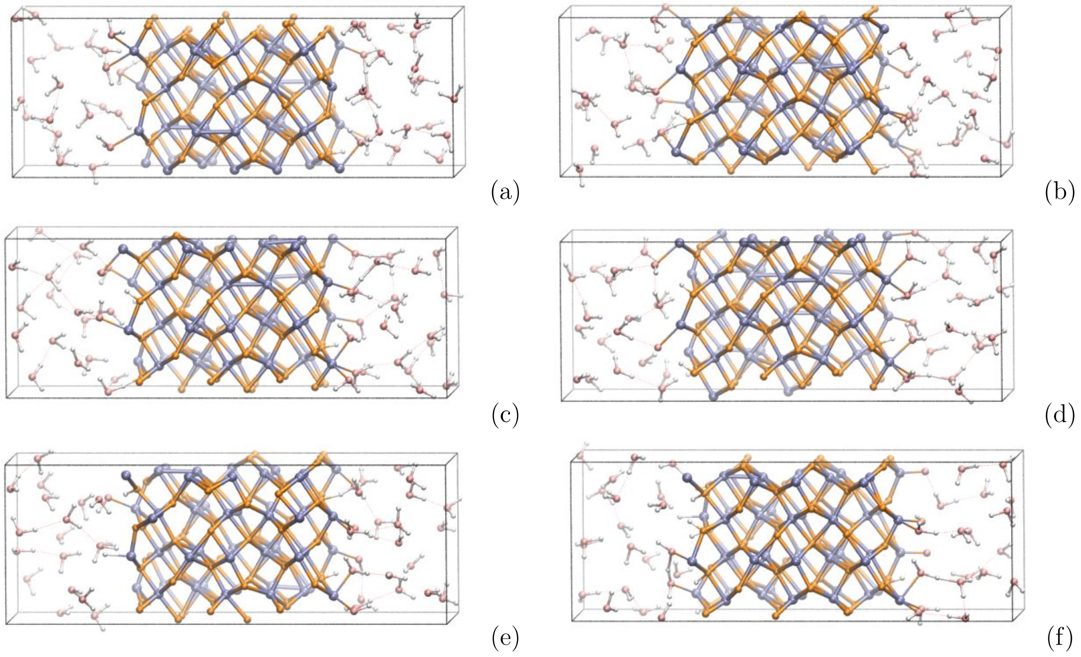

Figure S1: Snapshots of the model supercells under (a) zero-field conditions after equilibration, (b) zero-field conditions after 100 ps of production MD run, (c) applied field of 0.05 V/A magnitude at 87.4 ps, (d) field of 0.075 V/A at 99.2 ps, (e) field of 0.0875 V/A at 76.6 ps, and (f) field of 0.1 V/A at 51.3 ps. The hematite slab is in the centre of the box (Fe shown in light blue, oxygen in orange). As in Fig. 1 of the main manuscript, the applied-field direction is from left to right along the supercell (anti-parallel to the surface normal at the left-hand side, 'lower' interface, and parallel therewith in the case of the right, 'upper' interface).

Corresponding authors: * zfutera@prf.jcu.cz, niall.english@ucd.ie, Tel: +353-1-7161646 (NE), Fax: +353-1-7161177 (NE)

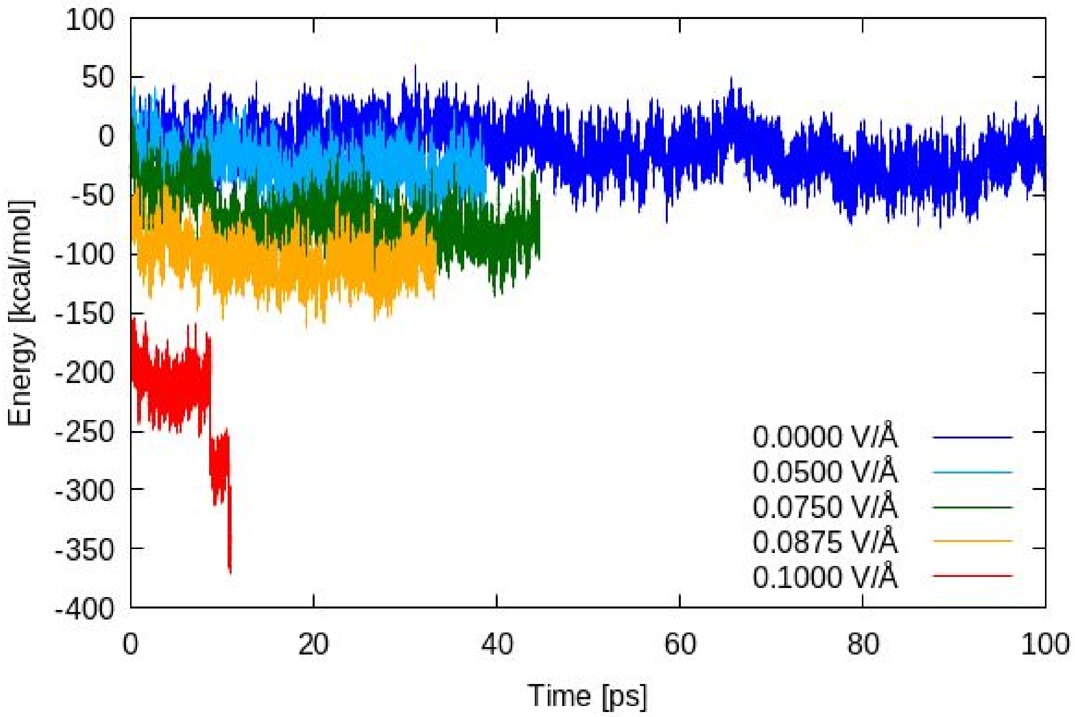

Figure S2: System potential energy during (NE-) AIMD simulations under zero-field conditions and external static electric fields of maginitude 0.05 V/Å, 0.075 V/Å, 0.0875 V/Å and 0.1 V/Å applied along the supercell in direction perpendicular to the hematite surface. The energy scale is relative to the mean value of the zero-field trajectory, which is designated here as nought. The shown values are from production AIMD runs following previous equlibration.

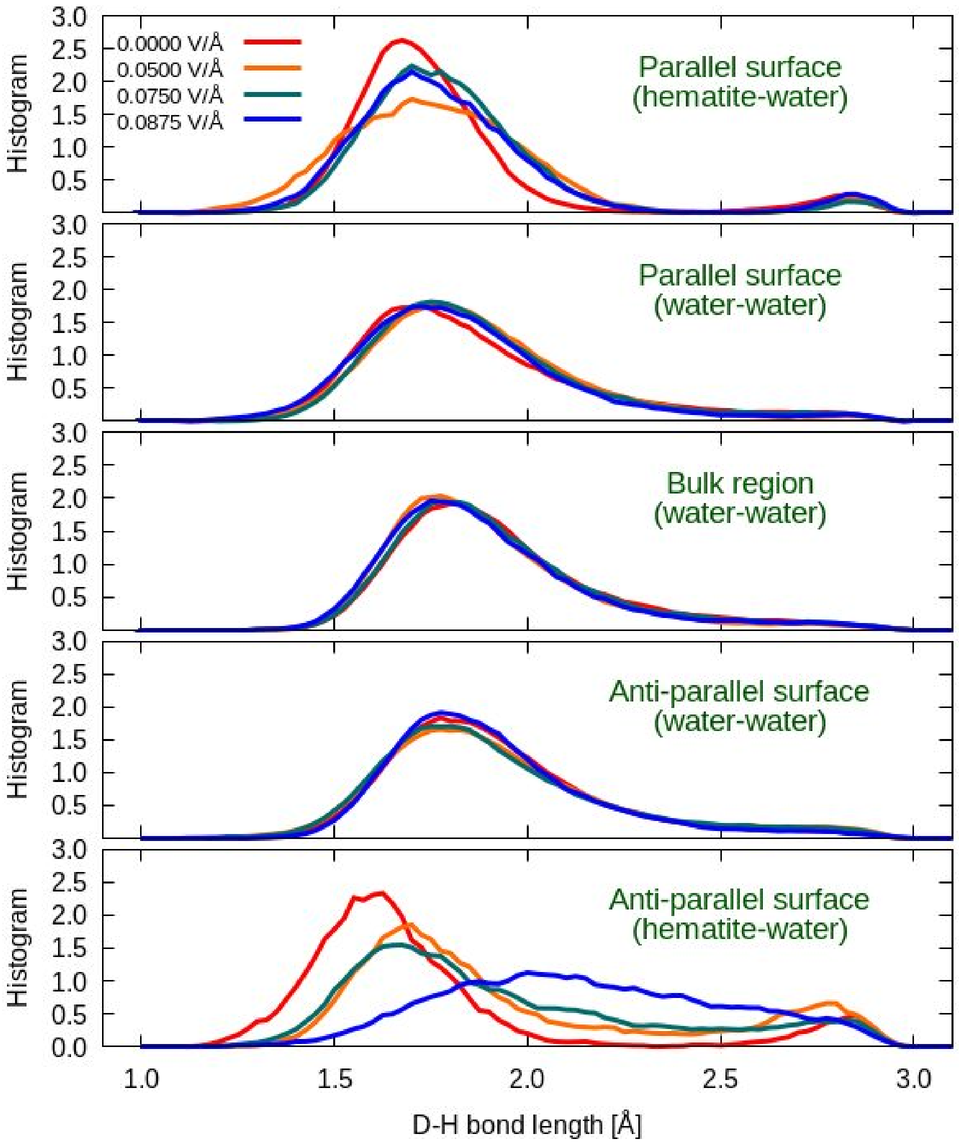

Figure S3: Distributions of hydrogen-bond lengths in water bulk region as well as at aqueous hematite interfaces where the applied static electric field is either parallel or anti-parallel to the surface normal (cf. Fig. 1 in the main manuscript for parallel alignment on RHS surface, and anti-parallel in the case of the LHS surface).

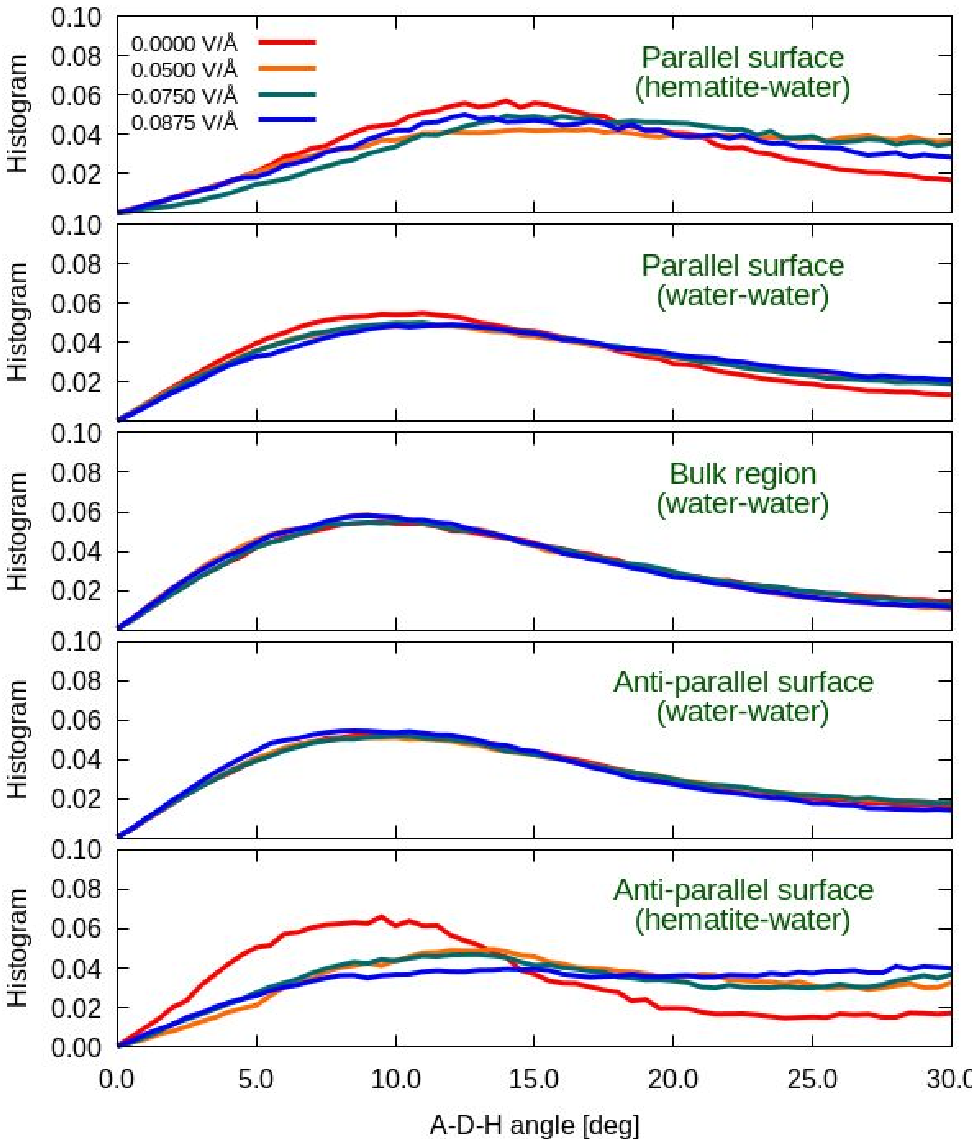

Figure S4: Distributions of hydrogen-bond A-D-H angles in water bulk region as well as at aqueous hematite interfaces where the applied static electric field is either parallel or anti-parallel to the surface normal (cf. Fig. 1 in the main manuscript for parallel alignment on RHS surface, and anti-parallel in the case of the LHS surface).

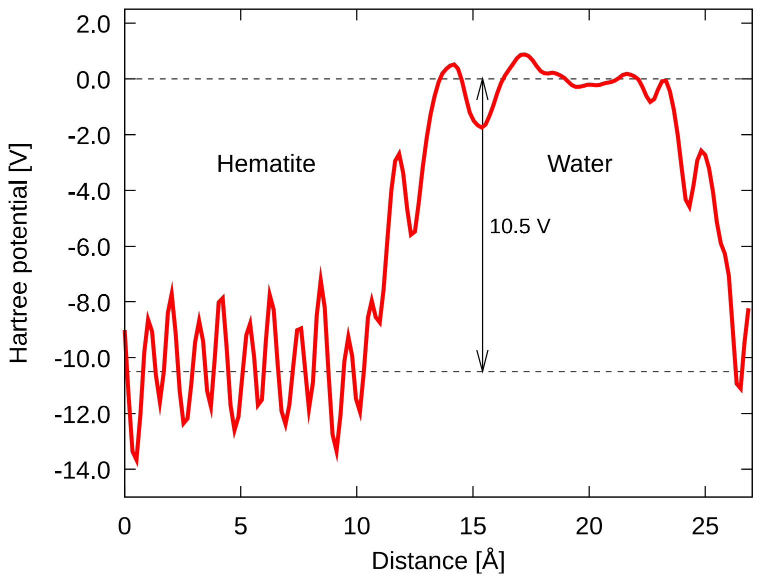

Figure S5: Mean Hartree potential $V_H(z)$ under zero-field conditions obtained as an average over 100 samples from AIMD trajectory. Potential profile is shown along the whole supercell and the mean values in hematite and water region are indicated by horizontal dashed lines.

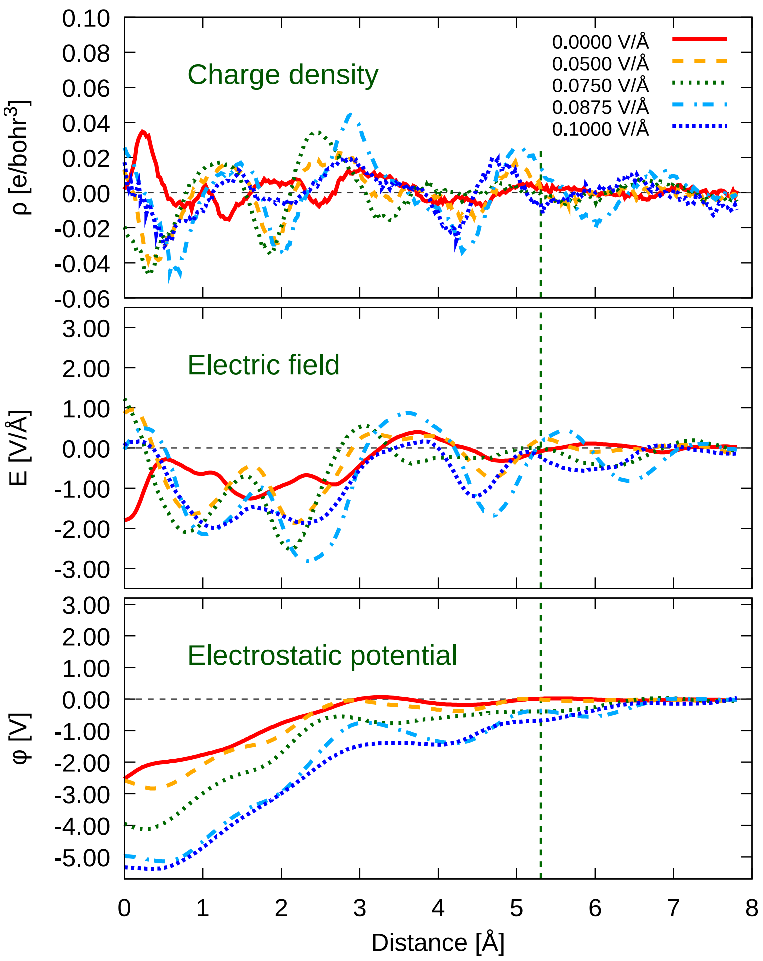

Figure S6: Charge distribution ρ(z) of Hirshfeld charges in the water region of the supercell as collected during (NE-)AIMD, and the corresponding electric-field E(z) and electrostatic-potential profiles φ(z). The distance is measured from the avarage position of the outermost Fe-atom layer at the hematite surface. The vertical dashed green lines mark the de-facto boundary of the interfacial region.
